# Inflammatory Biomarkers Profile as Microenvironmental Expression in Keratoconus

**DOI:** 10.1155/2016/1243819

**Published:** 2016-08-03

**Authors:** Catalina Ionescu, Catalina Gabriela Corbu, Cristiana Tanase, Christian Jonescu-Cuypers, Cristina Nicula, Dana Dascalescu, Miruna Cristea, Liliana-Mary Voinea

**Affiliations:** ^1^Clinical Hospital of Ophthalmologic Emergencies (SCUO), Piața Alexandru Lahovari 1, Bucharest, Romania; ^2^Division of Ophthalmology, Faculty of Medicine, “Carol Davila” University of Medicine and Pharmacy, Bulevardul Eroii Sanitari 8, 050474 Bucharest, Romania; ^3^Biochemistry-Proteomics Department, Victor Babes National Institute of Pathology, Sector 5, 050096 Bucharest, Romania; ^4^Faculty of Medicine, Titu Maiorescu University, Dâmbovnicului Street 22, 040441 Bucharest, Romania; ^5^Department of Ophthalmology, Charité University Hospital, Campus Benjamin Franklin, Hindenburgdamm 30, 12203 Berlin, Germany; ^6^Division of Ophthalmology, Faculty of Medicine, University of Medicine and Pharmacy “Iuliu Hatieganu” Cluj-Napoca, Victor Babes Street 8, 400012 Cluj-Napoca, Romania; ^7^Clinical Emergency Hospital Cluj-Napoca, Clinicilor Street 3-5, 400006 Cluj-Napoca, Romania; ^8^Department of Ophthalmology, University Emergency Hospital, Splaiul Independenței 169, 050098 Bucharest, Romania

## Abstract

Keratoconus is a degenerative disorder with progressive stromal thinning and transformation of the normal corneal architecture towards ectasia that results in decreased vision due to irregular astigmatism and irreversible tissue scarring. The pathogenesis of keratoconus still remains unclear. Hypotheses that this condition has an inflammatory etiopathogenetic component apart from the genetic and environmental factors are beginning to escalate in the research domain. This paper covers the most relevant and recent published papers regarding the biomarkers of inflammation, their signaling pathway, and the potentially new therapeutic options in keratoconus.

## 1. Introduction

Keratoconus is a progressive, degenerative, usually bilateral disease of the cornea that leads to refractive errors (myopia and irregular astigmatism) with impaired visual acuity and conical corneal protrusion [1]. The progressive thinning of the stroma is the main condition responsible for corneal ectasia. Keratoconus affects typically adolescents at puberty and young adults until the fourth decade of life with the age between approximately 12 and 35 years, during which the disease can progress or spontaneously arrest [1, 2]. The pathophysiology of keratoconus is multifactorial and is still not completely understood. There are proofs that biochemical, biophysical, and genetic aspects play an important role in the etiology of this ectatic corneal disorder. The familial inheritance and the high correlation among monozygotic compared to dizygotic twins [1] show that keratoconus has also a genetic component. Its association with multiple systemic and ocular disorders such as Down syndrome, Leber congenital amaurosis, or Ehler-Danlos syndrome is another aspect that supports this [3]. An interplay between environmental and genetic factors is convincing for the development of the disease. Contact lens wear and eye rubbing are two of the most important exogenous environmental aspects that induce mechanical changes causing corneal epithelial microtraumas that stimulate the expression of cellular inflammatory mediators [4].

Atopy is yet another studied risk factor that shows a correlation with keratoconus, although it is not well demonstrated whether the atopic ground itself or its effect (eye rubbing) is the one responsible for the effects on the cornea [5].

The most important aspect is the stromal degradation and its thinning, which has many hypotheses. Multiple studies relate the thinning to increased levels of proteolytic enzymes on the one hand and decreased levels of their inhibitors on the other hand [6].

The abnormal collagenolytic activity of the cells and the accelerated apoptosis of keratocytes induce a loss of extracellular matrix and redistribution of collagen fibrils. These actions result in stromal thinning and possible breaks in Bowman's layer with subsequent scarring [2, 7].

## 2. Histopathological Changes in an Injured Cornea

The corneal epithelium is a nonkeratinized, stratified, squamous 50 *μ*m thick tissue composed of 6-7 cell layers. It is covered by a tear film, which is responsible for lubricating and protecting the surface from microbial pathogens and foreign bodies [9]. Bowman's membrane is an acellular, nonregenerating layer composed of types I, III, V, and VI collagen fibrils. The stroma represents approximately 85% of the corneal thickness. This substance is formed mainly out of type I collagen with special distribution and parallel orientation of the lamellae. The stroma consists of proteoglycans (keratin sulfate, dermatan sulfate) and keratocytes that play a key role in stabilizing the extracellular matrix. The endothelium is composed of a single layer of hexagonal cells that secretes Descemet's membrane towards the stroma. The most inner layer of the cornea acts as a barrier between the stroma and the aqueous humor and permits a sufficient ion flux to maintain the osmotic gradient [10].

In case of an epithelial injury, cell membranes extensions cover the edge of the wound. Fibronectin, an extracellular matrix protein that mediates the cell adhesion and migration, plays a key role in wound healing [9, 11].

Gelatinase B (metalloprotease-9) could be a factor in delaying the normal process of epithelial defect healing [12]. After a stromal injury, the stromal keratocytes are activated by enlarging their size and transforming into fibroblast-like cells. After this process, the keratocytes engage in apoptosis and myofibroblasts begin to remodel the stroma on a background of inflammation with increased levels of growth factors, cytokines, and matrix metalloproteases [4]. The response of an injured endothelium is the enlargement and migration of the adjacent cells [9].

## 3. Histopathology of Keratoconus

The major clinical signs of keratoconus are thinning of the corneal stroma (mainly inferiorly, which gives birth to the characteristic conical shape), Fleischer's ring (a complete or incomplete circumferentially iron deposit), Vogt's striae in the deep stroma, and Descemet's membrane, followed by possible stromal scars, visible nervous fibers, and ruptures in Descemet's layer in a more advanced stage of the disease [1]. In a histological study performed by Scroggs et al., “typical” keratoconus corneas presented an important thinning of the epithelium centrally, breaks localized in Bowman's layer, and morphological modifications of the superficial epithelial cells [13, 14].

The stromal collagen lamellae in keratoconic corneas tend to have specific orientation compared to normal subjects and are reduced in number. There is a direct correlation between stromal thickness and quantity of collagen fibrils [15]. It has been proven that the morphology and density of keratocytes are altered in the anterior stroma in contact lens wearers diagnosed with keratoconus [16].

## 4. Keratocytes' Dysfunction in Keratoconus

Pouliquen et al. showed that keratocytes of keratoconic eyes have fourfold more IL-1 receptors compared with normal subjects [17]. The effects of IL-1 are activation of collagenases, metalloproteinases, and overexpression of both keratinocyte growth factor and IL-6 [18, 19]. IL-1 stimulates the KC fibroblasts and triggers a massive production of prostaglandin E2 and, in contrast, a low collagen production [20]. Wilson et al. observed in vitro that IL-1 alpha and IL-1 beta induce cell apoptosis in the stroma, leading to altered tissue organization in keratoconic patients [4]. Transforming growth factor-beta 1 has the capacity of differentiating corneal keratocytes into myofibroblasts in order to stabilize the tissue. The other aspect is the upregulation of inflammatory cytokines activated by TGF-beta, resulting in cellular apoptosis. An alteration of this pathway could lead to corneal fibrosis. These facts explain the hypothesis that TGF-beta 1 could play a role in the scar formation in keratoconic corneas [21].

## 5. Biomarkers of Inflammatory Pathway in Keratoconus

The International Programme on Chemical Safety defined a biomarker as “any substance, structure, or process that can be measured in the body or its products and influence or predict the incidence of outcome or disease”. It is a promising field and an accessible and minimally invasive method for early diagnosis and disease progression obtained from body fluids or tissue biopsies. As an example for their importance, researchers have discovered new protein and cytokines biomarkers that are overexpressed in glioblastoma patients compared to healthy subjects [22, 23].

Biomarkers aim at the early detection of cancer and the development of personalized treatment. Thus, biomarkers are becoming a priority in oncology. However, biomarker panels have proven to make a difference in the approach of keratoconus, taking into account the perspective of an inflammatory rather than a noninflammatory disorder.

An important role in the immune system is played by the T helper cells that have regulatory immune characteristics by releasing T cell cytokines, activating both cytotoxic T cells and macrophages. Th cells can be categorized in effector, memory, and regulatory T cells. While type 1 Th cells promote mostly the cellular immune system by stimulating the macrophages, type 2 Th cells are responsible for the humoral immune response through the proliferation of B cells, thus inducing the production of antibodies. Th 17 cell is yet another type of proinflammatory T cells and is involved in the production of interleukin-17, frequently associated with allergic responses. Among the proinflammatory mediators secreted by Th1 cells are interleukin-2, TNF (tumor necrosis factor), interferon-gamma, IL-12, and IL-15, while IL-4, IL-5, IL-9, IL-10, and IL-13 are released by Th2 cells [24].

Cytokines are glycoprotein molecules that are secreted by immune cells and can trigger an immune response through their complex interactions. These substances can initiate, amplify, or downregulate the immune response, as well as influencing cell proliferation and inflammatory processes, and are also called because of their actions inflammatory mediators [24].

T helper 1 cells are responsible for secreting interleukin-2 (IL-2) and interferon-gamma (IFN-gamma) that play an important role in the activation of macrophages and in the immune responses against intracellular pathogens. Th1 responses are connected to IL-12 that promotes the release of IFN-gamma and to IL-23 that enhances IL-17 release [25]. IL-4, IL-5, IL-10, and IL-13 are produced by T helper 2 cells that are closely related to allergic and antibody responses by triggering immune responses against extracellular pathogens. IL-6 is also produced by T helper 2 and stimulates both the cellular and humoral immune response. Th17 cells' role is the production of IL-17 family and of chemokine CCL20 that have a strong component in chronic tissue inflammation [26]. Interleukin-1 family contains 11 members that are mainly produced by monocytes, fibroblasts, and macrophages and expressed by B lymphocytes and have an important role in mediating the inflammation. IL-1 alpha, IL-1 beta, and the IL family of ligands and receptors are strongly connected in the process of apoptosis and necrosis in inflammation [27].

The most important aspect of these signaling inflammatory molecules is the balance between them with a positive or negative regulatory interaction. In acute or chronic diseases, Th 1 and Th 2 actions are amplified; thus, an increase or decrease of some cytokines can be detected and monitored. For example, IFN-gamma and IL-12 stimulate Th1 but inhibit proliferation of Th 17 cells [24]. Th2 (IL-4 and IL-10) has an inhibitory action on Th1 cells through a decreased production of IL-12 [28].

The cytokines have many characteristics: they are pleiotropic (different effects on different tissues) but also redundant (when different types of cytokines react in the same matter). These low molecular weight proteins have the ability on the one hand to antagonize each other and on the other hand to respond with a positive feedback to other cytokines [29].

Tumor necrosis factor-alpha is a transmembrane protein produced by macrophages, lymphoid cells, and fibroblasts in response to bacterial products, IL-1 or IL-6. TNF-alpha is considered a significant mediator of systemic and local inflammation. Recent studies show that the inflammatory activity of TNF and TNF ligand family is more important than their role in apoptosis [30].

Matrix metalloproteinases are a type of enzymes regulated by cytokines (IL-1, IL-6, and IL-7), TNF-alpha, growth factors, and hormones that play a deciding role in the degradation of extracellular matrix proteins and in cell proliferation and apoptosis. MMPs are inhibited by tissue inhibitor of metalloproteinases (TIMP) [31]. This complex (MMP-TIMP) is responsible for the integrity of the connective tissue and a normal wound healing after injuries [32].

## 6. Microenvironmental Changes and Cytokines Signaling in Keratoconus

Keratoconus was first described as a noninflammatory ectatic disease, a theory that is beginning to be contradicted by multiple studies that have brought strong evidences for sustaining the clause for the role of inflammation in the pathogenesis of the ectasia. The studies mentioned below will highlight the most relevant conclusions concerning the topic of inflammation in keratoconus.

In 2009, Lema et al. proved the possible inflammatory pathogenesis of keratoconus, showing an increased level of IL-6 and TNF-alpha in subclinical and keratoconic eyes, while MMP-9 was detected only in tears of the patients with the manifest disease [33].

Sorkhabi et al. demonstrated the marked presence of proinflammatory cytokines in the tear fluid in 42 subjects with keratoconus, such as IL-6, IL-1 beta, and interferon-gamma, and a decreased level of the anti-inflammatory IL-10 [34].

Few studies reflected the correlation between inflammatory mediators in the tear fluid and the severity of keratoconus. Kolozsvári et al. studied the correlation between inflammatory cytokines in the tear fluid and the severity of keratoconus. They revealed a significant positive association between CCL5 (chemokine ligand 5) and center/surround index as well as between IL-6 and maximum K value, yet a negative one between IL-13 and the severity of the disease. The researchers also found increased levels of nerve growth factor in keratoconic patients [35].

In the latest study, Pásztor et al. observed the association between cytokines and some Pentacam parameters and found strong positive correlations between CXCL8 and BAD-D (Belin-Ambrosio deviation index). MMP-9 levels were significantly increased in association with BAD-D and K2 keratometry values [36].

An intense proteolytic activity causes collagen denaturation that may accelerate the progression of the disease. Balasubramanian et al. studied the importance of proteolysis in the progression of keratoconus by comparing the total tear protein level, the protease, and inflammatory molecules in patients with keratoconus, patients after corneal collagen cross-linking, and normal subjects. The study showed increased levels of gelatinases and collagenases (1.9 times higher), as well as elevated MMPs, cytokines (MMP-1, MMP-3, MMP-7, MMP-13, and IL-6), and TNF-alpha and TNF-beta in the keratoconus group compared with the normal group [37]. In another study, the researchers observed a significant increase in the tear film of keratoconic patients of cathepsin B level, a lysosomal protease capable of degrading extracellular matrix proteins. On the other side, there were downregulated levels of cystatin (a group of protease inhibitors) [38]. One year later, Balasubramanian et al. completed the previous study with a new hypothesis that eye rubbing modifies the tear levels of proteases and cytokines. A 60-second eye rubbing technique typically used by keratoconus patients had as a result a significant increase of MMP-13 in tears that has an important role in the apoptotic activity of the keratocytes. Also, TNF-alpha and IL-6 can also be found in atopic and vernal keratoconjunctivitis, suggesting that keratoconus could be related to allergies and to increased level of serum IgE [39].

Cheung et al. investigated the effect of injury in stromal cells in keratoconic corneas compared with normal subjects in order to determine whether there is a dysregulation in the reparative response. As a result, increased levels of IL1 alpha, TNF-alpha, and TGF-beta 1 were measured in keratoconic corneas without induced secondary injury compared with normal corneas and decreased levels of IL1 alpha, FGF-2, TNF-*α*, EGF, TGF-*α*, and PDGF were found in patients with keratoconus with secondary injury in relation to those without, which could emphasize the hypothesis of an ineffective wound healing in this ectatic disorder [40].

A presumed progression risk factor of keratoconus is the contact lens wear, especially rigid gas permeable contact lenses that induce the upregulation of IL-6, TNF-alpha, ICAM-1, and VCAM-1 in the tears of subjects with keratoconus [41].

Fodor et al. reported the theory of keratoconus' progression caused by contact lens wear that exacerbates the release of inflammatory mediators. The study observed increased levels of IL-6, MMP-9, and CXCL8 after contact lens wear and a decrease of nerve growth factor, TIMP1, and PAI1 (principle inhibitor of tissue plasminogen activator). The study revealed that this can alter the stromal structure through matrix degradation and proapoptotic effect [42].

In normal subjects, lactoferrin downregulates the expression of cytokines and proteinases. Lema et al. discovered in 2010 in patients with keratoconus the underexpression of lactoferrin, an antimicrobial and anti-inflammatory protein, that suggests a disruption of the protective barrier [43]. Chaerkady et al. made a complete proteome analysis of the cornea in keratoconus and observed overexpression of keratins, extracellular matrix proteoglycans, and types I, III, and V collagen fibrils, yet a downregulation of lactotransferrin, which plead in favor of a degenerative and inflammatory disease. They studied the corneal proteome in keratoconus and identified 932 proteins in the epithelium, respectively, 1,157 in the stroma, that brought to light the resemblance with other neurodegenerative disorders but also the inflammatory component and the importance of oxidative stress [44].

Pannebaker et al. published statistically significant increased levels of MMP-1 only in keratoconic eyes. They also studied tumor necrosis-related apoptosis-inducing ligand-R1 (TRAIL-R1) that had a decreased level in the gas permeable lens wearer keratoconus group and increased in the one without lenses. These results could suggest the alteration of the receptors in keratoconus [45].

Allergic states are correlated with increased levels of cytokines in the tears of patients with keratoconus. Weed et al. reviewed in their article the strong correlation between keratoconus and ocular allergy, as well as the positive association with atopy (asthma, eczema, and hay fever) [46]. Because allergy is a risk factor of keratoconus, Sharma et al. suggested that corneal topography should be a routine investigation in these patients [47]. Bawazeer et al. concluded that the most important risk factor of the disease is eye rubbing that could be induced by the itch of atopy [5].

Kolozsvári et al. analyzed the evolution of tear's biomarkers in keratoconic patients after corneal collagen cross-linking and reported that the levels of IL-6 and IL-8 decreased 1 year after CXL. The study revealed also a negative association between IL-6 and Th1, respectively, between MMP-13 and keratoconus index (KI) [48]. Analyses of the tear fluid showed abnormal levels of TH1, TH2, and TH17 cytokines, suggesting certain immune dysregulation in this disease as well [49].

Jun et al. analyzed the levels of Th1, Th2, and Th17 cell cytokines in the serum and tears of keratoconic patients in order to correlate a systemic inflammation with keratoconus. There were no significant levels of proinflammatory serum cytokines, in contrast to increased levels of IL-6 and IL-17 in the tear film of keratoconus patients. IL-4, Il-12, IL-13, and TNF-alpha were found to be downregulated in the keratoconus group [50].

More recent studies evidence the correlation between the progression of keratoconus and a systemic inflammation through a new measure called neutrophil-to-lymphocyte ratio (NLR). Karaca et al. found that the NLR was higher in the patients that presented progression opposed to the stationary and normal subjects [51].

**Figure 1:**
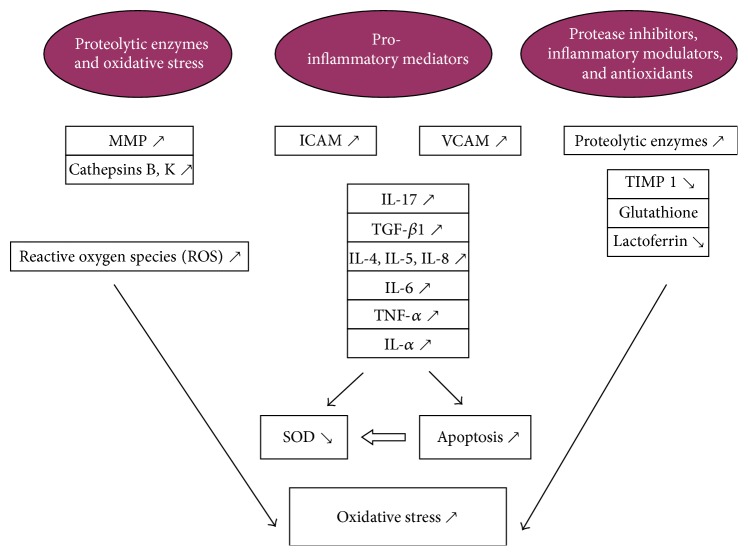
Adapted by Galvis et al. [8], illustrating the interplay between cytokines, proteases, and antioxidants and their complex multidirectional actions.

There is a vicious circle between the proinflammatory cytokines, proteolytic enzymes, and inhibitors that are the ones responsible for the microenvironmental changes in keratoconus. This imbalance triggers the signaling of inflammatory pathways in the cornea inducing structural abnormalities that lead to progression of the disease (Figure 1) [8].

## 7. Enzymatic Profile in Keratoconus

MMP-9 is a gelatinase that belongs to the metalloproteinases family and is responsible for degrading the denatured collagen fibrils. Multiple studies have shown a positive correlation between MMP-9 and a multitude of diseases, such as keratoconus, herpetic keratitis, and Sjogren's syndrome, which all have an inflammatory component [52].

In keratoconus, MMPs are found to be overexpressed in every corneal structure, while TIMP levels are decreased, suggesting the hypothesis of tissue degradation. Lema et al. put the emphasis on subclinical keratoconus, comparing it to manifest keratoconus. They observed increased levels of MMP-9, tumor necrosis factor, and interleukin-6 in the tear film of patients with keratoconus and overexpression of IL-6 and TNF-alpha in the tears of subclinical keratoconic patients. As a conclusion of this study, they demonstrated the possibility of a significant progression risk towards bilateral disease [33]. Seppala et al. showed through immunohistochemical labeling that the extracellular matrix metalloproteinase inducer CD147 and MMP-1 are overexpressed in keratoconus, being responsible for degrading the fibronectin, membrane glycoproteins, and types I and III collagen [53].

Collier focused on immunohistochemistry and observed that MMP-14 had increased levels in the corneal epithelium and stroma. MMP-14 could overexpress MMP-2, thus activating the digestion of type IV collagen lamellae [54].

Mackiewicz et al. labeled corneal enzymes that have a potential of digesting collagen and observed first of all MMP-13 upregulation and a high presence of cathepsin K and human trypsin-2 in keratoconic patients compared to the control group. Once again, the importance of gelatinase A (MMP-2) and MMP-14 in corneal remodeling and the fact that increased levels of those enzymes could be a sign of deficient healing were ascertained [55].

## 8. Dysregulation of Oxidative Status in Keratoconus

A key role of the cornea is to neutralize free oxygen radicals and oxidants that are produced constantly by ultraviolet light and cellular metabolites. Oxidative stress begins to gain importance in the pathophysiology of glaucoma, age related macular degeneration, retinopathy of prematurity, and keratoconus [56, 57]. The main factors that protect the ocular tissue against oxidative damage are superoxide dismutase (protection against superoxide radicals), low molecular weight antioxidants such as ascorbic acid, ferritin, and glutathione, and high molecular weight antioxidants (catalase and glutathione peroxidase) [8, 57].

In order to predict the oxidative stress, the total oxidant and antioxidant status are measured, as well as the ratio OSI (oxidative stress index) between these two parameters [58]. Toprak et al. were the first ones to reveal that oxidative stress could be a predisposing factor for keratoconus. A higher OSI is an indicator of progression of keratoconus [59]. The balance between the formation of free radicals and their removal by antioxidants is altered in keratoconic corneas, which have a lower content in glutathione. Therefore, the final outcome is an accumulation of aldehydes and peroxynitrites that have a destructive, cytotoxic effect on the tissue [60, 61]. Olofsson and coworkers demonstrated that the upregulation of interleukin-1 alpha reduces the synthesis of superoxide dismutase and harms the normal antioxidant barrier [62]. Kenney et al. concluded that overexpression of cathepsins triggers the production of hydrogen peroxide. A decreased level of TIMP-1 that has antiapoptotic actions is responsible for the destruction of stromal architecture. TIPM-3 however has proapoptotic characteristics. This imbalanced ratio could shift the effect in favor of keratocytes apoptosis in keratoconic corneas [63].

## 9. Therapeutic Options in Keratoconus

Optical correction in early stages of the disease can be achieved with spectacles, with soft contact lenses, or in more advanced stages with rigid gas permeable, scleral, or hybrid lenses [64]. Another therapeutic minimal invasive option is collagen cross-linking that aims to halt the progression of keratoconus by increasing the collagen fibrils' rigidity using riboflavin as photosensitizer and UVA light. Conventional protocol at 3 mW/cm^2^ for 30 min (5.4 J/cm^2^ energy dose) releases reactive oxygen species (ROS) that induces corneal stiffness. Another way is to shorten the duration of the procedure by increasing the intensity. Both are continuous light treatments. Recent studies emphasize the important role of tissue oxygenation, thus introducing the new protocol of pulsed light accelerated cross-linking [65].

Beside the minimal invasive therapy by corneal cross-linking, there are also surgical treatment modalities in keratoconus. In patients with advanced disease, it may be necessary to perform corneal transplantation, either a deep anterior lamellar keratoplasty (DALK) or penetrating keratoplasty (PK). DALK is a procedure based on a perfect dissection plane between the Descemet membrane and the deep stromal layer using basic salt solution or air in order to remove the corneal stroma below the Descemet membrane. In advanced stages of keratoconus is penetrating keratoplasty, which is indicated if Descemet membrane's or the corneal epithelium's integrity is altered. Intrastromal corneal ring segments (ICRSs) represent also an option in keratoconus. These are made of PMMA (polymethyl methacrylate) and are implanted in the deep corneal stroma in order to modify the corneal curvature [2, 66].

Kenney et al. proposed in his study the potential benefit of using ultraviolet light protection in order to prevent the oxidative mechanism that could have a negative impact on the progression of keratoconus [67]. Another hypothesis concerning new therapeutic management in keratoconus is the one suggested by Cheung et al., according to which riboflavin could have a constructive effect on the extracellular matrix and downregulate ROS [68].

Regarding the possible inflammatory etiology of the disease, Shetty et al. proposed Cyclosporine A, an immunosuppressant drug with strong anti-inflammatory characteristics, as a potential therapeutic option in keratoconus. In this study, 27 eyes with increased inflammatory biomarkers in the tear film were treated with topical Cyclosporin A. After 6 months, a clear downregulation of MMP-9, IL-6, and TNF-alpha was observed, as well as an important local flattening and reduction of corneal curvatures measured by corneal topography [69].

Priyadarsini et al. investigated the TGF-beta signaling in the pathogenesis of keratoconus on isolated human keratoconic cells from patients with advanced disease who underwent corneal transplantation. TGF-*β*1, TGF-*β*2, and TGF-*β*3 are TGF-*β* isoforms with a key role in extracellular matrix reorganization, keratocytes' differentiation to myofibroblasts, and activation of matrix metalloproteinases. While TGF-*β*1 and TGF-*β*2 have a profibrotic activity as a reaction to an injury, TGF-*β*3 is responsible for the antifibrotic effect. The study showed that TGF-*β*3 has the capacity to lower the levels of the key receptor TGF-*β*RII and as a result to ameliorate the profibrotic component of keratoconic human cells. Very important elements of the TGF-*β* pathway are the SMAD proteins, which are modified in keratoconus, thereby altering the signaling that could lead to accentuated fibrosis of corneal tissue in the process of wound healing. Priyadarsini et al. suggest that control and regulation of TGF-*β* receptor could be a new therapeutic option in the treatment of keratoconus [70].

## 10. Conclusion

The pathogenesis of keratoconus is still poorly understood. Until a few years ago, keratoconus has been defined as a degenerative, noninflammatory disease due to the absence of both corneal neovascularization and inflammatory cells infiltration. Mcmonnies explained in his study the consequences of eye rubbing in patients with keratoconus. The rubbing related corneal trauma could increase the corneal temperature, overexpress the levels of proinflammatory cytokines and proteinases in the tear film, and cause epithelial thinning with repercussions on every layer of the cornea [71].

In the future, the tear proteomics in keratoconus will be studied intensively to identify specific biomarkers for prevention or early diagnosis and new therapeutic options. We have now the information to state that keratoconus is a complex disease with a multitude of factors including genetic, environmental (external), and microenvironmental components.

We conclude that a key role in the pathogenesis of keratoconus is the altered balance between inflammatory cytokines, proteases, and proteases inhibitors, as well as free radicals and oxidants [8].

After reviewing the most relevant and recently published results, we emphasize the contribution of the altered signaling pathway of proinflammatory mediators in the pathogenesis of keratoconus and their role in the disease progression. The measured interleukins and metalloproteinases are biomarkers, although not sensitive nor specific for keratoconus. There are ongoing studies that try to identify a specific biomarker for early detection of the disease. In the future, such biomarkers could improve the therapeutic outcome [72].
